# Concurrent Improvement Observed in Patient-Reported Burden and Sensor-Collected Medication Use Among Patients Enrolled in a COPD Digital Health Program

**DOI:** 10.3389/fdgth.2021.624261

**Published:** 2021-04-09

**Authors:** Leanne Kaye, Rahul Gondalia, Meredith A. Barrett, Melissa Williams, David A. Stempel

**Affiliations:** ^1^ResMed Science Center, San Francisco, CA, United States; ^2^Propeller Health, San Francisco, CA, United States

**Keywords:** chronic obstructive pulmonary disease, COPD assessment test, digital health, medication adherence, short-acting beta-agonist, electronic medication monitoring

## Abstract

**Background:** The COPD assessment test (CAT) is an 8-item questionnaire widely used in clinical practice to assess patient burden of disease. Digital health platforms that leverage electronic medication monitors (EMMs) are used to track the time and date of maintenance and short-acting beta-agonist (SABA) inhaler medication use and record patient-reported outcomes. The study examined changes in CAT and SABA inhaler use in COPD to determine whether passively collected SABA and CAT scores changed in a parallel manner.

**Methods:** Patients with self-reported COPD enrolled in a digital health program, which provided EMMs to track SABA and maintenance inhaler use, and a companion smartphone application (“app”) to provide medication feedback and reminders. Patients completing the CAT questionnaire in the app at enrollment and at 6 months were included in the analysis. Changes in CAT burden category [by the minimally important difference (MID)] and changes in EMM-recorded mean SABA inhaler use per day were quantified at baseline and 6 months.

**Results:** The analysis included 611 patients. At 6 months, mean CAT improved by −0.9 (95% CI: −1.4, −0.4; *p* < 0.001) points, and mean SABA use decreased by −0.6 (−0.8, −0.4; *p* < 0.001) puffs/day. Among patients with higher burden (CAT ≥ 21) at enrollment, CAT improved by −2.0 (−2.6, −1.4; *p* < 0.001) points, and SABA use decreased by −0.8 (−1.1, −0.6; *p* < 0.001) puffs/day.

**Conclusion:** Significant and parallel improvement in CAT scores and SABA use at 6 months were noted among patients enrolled in a digital health program, with greater improvement for patients with higher disease burden.

## Introduction

Chronic obstructive pulmonary disease (COPD) is a progressive respiratory illness with substantial impact on the patient's well-being (1). The COPD Assessment Test (CAT) is a validated questionnaire designed to assess the disease burden (2). While the CAT is widely accepted in clinical practice, administration is not performed at any routine cadence, limiting its clinical value to identify patients with declining disease status potentially needing intervention.

Remote patient monitoring (RPM) with digital health tools for COPD may enhance the current standard of care through regular monitoring of symptoms and medication-taking behaviors (3, 4). Today, there exists an abundance of digital tools to support RPM, including wearables, smartphone or mobile phone applications, short-messaging services (SMS), and sensors to track medication use. These tools may help collect data between office visits and provide regular insight into patient health.

In COPD, digital tools may also help enhance care through recording use of as-needed inhaled short-acting beta-agonists (SABA) for symptoms, monitoring adherence to daily maintenance inhaler medication, and measuring lung function. Electronic monitoring of SABA use may help identify patients who have acute worsening of symptoms outside of routine provider visits (5–7). Although SABA use is not captured in CAT, symptoms that are commonly treated with SABA are a major component of the questionnaire.

This study assessed changes in CAT scores and SABA use over 6 months among patients with COPD enrolled in a digital self-management platform, which included electronic medication monitors (EMMs) and a smartphone application (“app”).

## Methods

**Figure 1:**
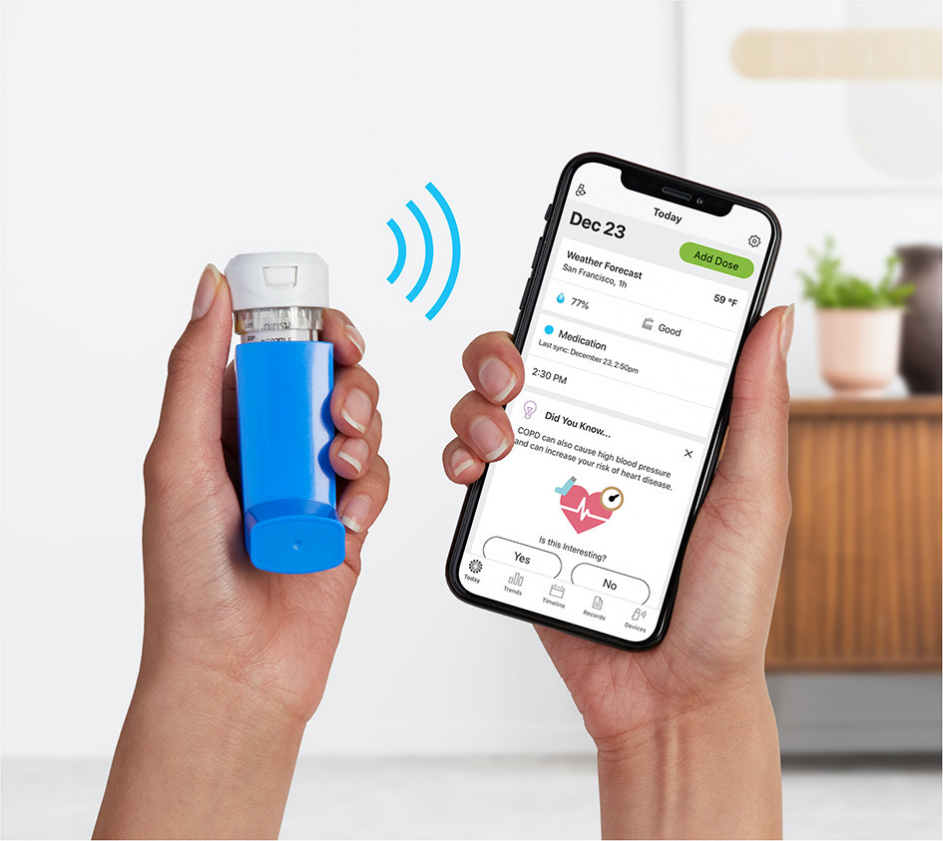
A small electronic medication monitor (EMM) attaches to a metered dose inhaler (MDI) to collect data on inhaler usage. Data is then transmitted wirelessly via Bluetooth to a paired patient-facing smartphone application.

Patients with self-reported COPD enrolled in a digital self-management platform (Propeller Health, Madison WI) between August 2017 and December 2019. Those with an EMM-compatible SABA inhaler and smartphone were eligible for the study. Patients were instructed to attach a small EMM to their SABA inhaler(s), and if available, to their maintenance inhaler(s) (Figure 1).

EMMs had an expected battery life of 12–18 months and did not require charging during the study period (8). The EMMs passively monitored the date and time of inhaler actuations when the EMM was depressed (6). These data were transmitted wirelessly via Bluetooth synchronizations (“syncs”) to the patient's smartphone app. Patients received evidence-based education, feedback on medication use, and reminders for scheduled medications and were prompted to complete an in-app CAT questionnaire monthly (2). Patients had the option to share their information with their providers but were not required to do so. To participate, patients were required to accept Propeller's Terms of Service which specified use of de-identified, aggregated data for publication (9). This retrospective analysis was reviewed by the Copernicus Institutional Review Board (PRH1-18-132).

Patients completing the CAT within 2 weeks from the date of the first EMM sync (baseline) and again at 6 months (152–212 days) from the date of the first sync were studied. CAT score burden was characterized as low (0–10), medium (11–20), high (21–30), and very high according to standard categories (31–40) (10). The first week of syncing was considered a platform learning period, and thus inhaler use during this period was excluded. Mean daily SABA use (puffs/day) and daily maintenance adherence (percent of puffs taken/prescribed, capped at 100%) were calculated during the 30 days following the baseline CAT and 30 days prior to the 6-month CAT. At baseline, unadjusted comparisons between mean daily SABA use and mean daily maintenance inhaler adherence by CAT burden were evaluated using a Wilcoxon rank-sum test (11). From baseline to 6 months, minimally important differences of CAT (2 points) and changes in CAT burden categories (low: CAT 0–10, medium: CAT 11–20, high: CAT 21–30, and very high: CAT 31–40) (2) were evaluated using the Chi-square test (12). Changes in CAT, mean daily SABA use, and mean daily maintenance adherence were estimated from baseline to 6 months using linear mixed-effect models (13) accounting for within-patient variability and adjusting calendar month to account for potential seasonal variation. Analyses were then stratified by lower (CAT < 21) vs. higher (CAT ≥ 21) burden categories to ensure a sufficient sample size. Because patients served as their own control, adjustment for static individual-level characteristics (e.g., age, gender) was not necessary. However, we did conduct sensitivity analyses to adjust for differences by age, CAT score, mean daily SABA use, and mean daily maintenance adherence (10). All statistical tests were two-tailed with an α = 0.05 threshold for statistical significance. All analyses were conducted in R version 3.4 (R Foundation for Statistical Computing).

## Results

The analysis included 611 patients {mean [standard deviation (SD)] age: 62 (8) years; 64% were >60 years}. All patients had a SABA inhaler EMM, and 371 (60.7%) also had a maintenance inhaler EMM. At baseline, mean (SD) CAT score was 22.6 (7.8). Mean (SD) SABA use was 2.3 (3.1) puffs/day, and mean (SD) daily adherence was 81 (27.1)%. Among patients with higher CAT burden, 60% were ≥60 years of age compared to patients with lower CAT burden where 71% were ≥60 years of age. Baseline SABA use was greater among patients with higher burden CAT scores compared to patients with lower burden CAT scores [median (IQR): 1.4 (0.4, 3.9) vs. 0.8 (0.1, 2.4) puffs/day; Wilcoxon rank-sum test *p* < 0.001], while baseline maintenance adherence was consistent [median (IQR): 93.3 (72.5, 98.3)% vs. 93.3 (80.7, 100.0)%; Wilcoxon rank-sum test *p* = 0.08].

At 6 months, 277 (45%) patients had CAT scores that improved by the minimally important difference (14). Using the MID as a minimum threshold, 154 (25%) patients moved from a higher burden category to lower burden category, while 351 (57.4%) patients had no category change. A larger percentage of patients in the higher burden group moved to a lower burden category or improved their CAT by the MID compared to those in the lower burden group (34.7 vs. 9.9%, Chi-square *p* < 0.001) (Table 1).

**Table 1:** Change in CAT burden category^a^ and MID change over 6 months using a Chi-square test.

	**Overall**	**Higher burden**	**Lower burden**	*P*-value^**^
Category improvement and MID decrease, *n* (%)	154 (25.2)	131 (34.7)	23 (9.9)	<0.001
Category worsening and MID increase, n (%)	106 (17.3)	40 (10.6)	66 (28.3)	
No change, *n* (%)	351 (57.4)	207 (54.8)	144 (61.8)	

a *Burden categories were defined as low (CAT 0–10), medium (CAT 11–20), high (CAT 21–30), and very high (CAT 31–40) burden*.

** *P-value derived from a Chi-square test comparing CAT burden category change in patients with higher vs. lower burden COPD*.

Linear mixed-effect models found that, from baseline to 6 months, mean CAT improved by −0.9 (95% CI: −1.4, −0.4; *p* < 0.001) points, mean SABA use decreased by −0.6 (95% CI: −0.8, −0.4; *p* < 0.001) puffs/day, and mean adherence decreased by −4.0 (95% CI: −6.9, −1.2; *p* < 0.01) percent. Among patients with higher CAT burden scores at enrollment, CAT improved by −2.0 (95% CI: −2.6, −1.4; *p* < 0.001) points, and SABA use decreased by −0.8 (95% CI: −1.1, −0.6; *p* < 0.001) puffs/day (Table 2). Sensitivity analyses did not change the observed outcomes significantly (Supplementary Table 1).

**Table 2:** Change in CAT, SABA use and adherence over 6 months using linear mixed effects models.

	**Mean (SD) at** ** baseline**	**Mean (SD) at** ** 6 months**	Estimates^*^	Lower 95% CI^*^	Upper 95% CI^*^	***P*-value**
**Overall**, ***n*** **=** **611**						
CAT	22.6 (7.8)	21.7 (8.1)	−0.9	−1.4	−0.4	*p* <0.001
SABA use, puffs/day	2.3 (3.1)	1.6 (2.5)	−0.6	−0.8	−0.4	*p* < 0.001
Adherence, %	81.1 (27.1)	76.8 (29.8)	−4.0	−6.9	−1.2	*p* = 0.01
**High burden**, ***n*** **=** **378**						
CAT	27.4 (4.7)	25.4 (6.6)	−2.0	−2.6	−1.4	*p* < 0.001
SABA use, puffs/day	2.7 (3.4)	1.8 (2.5)	−0.8	−1.1	−0.6	*p* < 0.001
Adherence, %	79.6 (27.0)	74.5 (30.3)	−4.6	−8.4	−0.9	*p* = 0.01
**Low burden**, ***n*** **=** **233**						
CAT	14.7 (4.8)	15.6 (6.5)	0.9	0.1	1.7	*p* = 0.03
SABA use, puffs/day	1.7 (2.4)	1.3 (2.3)	−0.3	−0.6	−0.1	*p* = 0.01
Adherence, %	82.8 (27.2)	80.0 (28.8)	−3.5	−8.2	1.2	*p* = 0.14

* *Adjusted for enrollment month*.

## Discussion

This study examines 6-month changes in CAT scores, SABA use, and maintenance adherence among patients using a digital self-management platform for COPD. Patients with higher COPD burden (CAT ≥ 21) had greater improvement with a significant and parallel reduction in both CAT and EMM-measured SABA use at 6 months. Moreover, many patients had a clinically meaningful change in CAT score. The concurrent reductions in CAT scores and SABA use suggest that passively collected SABA data may serve to highlight patients at risk of increasing COPD burden. Adherence to maintenance inhalers at baseline was high (80%) with only a 4% decrease over 6 months and not reflective of changes in CAT.

Digital technologies have demonstrated value in the clinical setting for patients with COPD (6, 7, 15). Alshabani (7) found that integration of an EMM with feedback in a 12-month quality improvement study resulted in earlier identification of patients with worsening condition, and significant reductions in SABA use. Similar outcomes were observed in 190 Medicare-eligible patients with COPD (6) where SABA use halved following a 6-month digital health intervention, which included patient outreach from clinical staff. Electronic monitoring of SABA use has also been identified as a potential predictor of exacerbations (5, 16) and higher SABA use has been associated with greater CAT burden (10).

Continued research is needed to better understand the mechanisms of behavior change and identify the most efficacious digital modalities. Programs integrating and/or based on behavior theories typically demonstrate stronger efficacy than those that do not (17). Further, programs relying on digital tracking alone may be less effective due to high attrition rates (18). Hybrid programs integrating both digital and human touch, as well programs including a combination of digital modalities (e.g., EMM plus smartphone app), have demonstrated stronger outcomes.

This study supports the use of a digital self-management platform to complement standard of care, but there were limitations. First, patients self-enrolled and were possibly more motivated to modify their behavior and health. Second, patients self-reported their COPD diagnosis, which was not confirmed given the remote design. Moreover, we did not have access to patient characteristics like gender and disease stage, which may limit the generalizability of the results observed. Lastly, while improved CAT and SABA use were observed over the study period, modest decreases in adherence were also detected. We hypothesize that improvement in COPD burden may have led patients to ease use of their maintenance inhaler; however, further investigation is needed to understand these behavioral changes.

## Conclusion

Patients enrolled in a digital self-management platform for COPD demonstrated significant and clinically meaningful improvement in CAT and a concurrent reduction in SABA use, especially among those with higher COPD burden. This study highlights the benefit of digital tools like EMMs to identify associations not observed with pre-digital modalities (self-report, prescription fills, etc.). However, EMMs are one tool in a myriad of consumer and medical-grade digital tools today. Future studies in COPD should explore the added value of digital platforms and/or tools that complement patient care by improving patient–provider communication, and better understanding chronic disease management.

## Data Availability Statement

The datasets presented in this article are not readily available because the medication use data that supported this study are not publicly available because they are considered Protected Health Information (PHI) under the Health Insurance Portability and Accountability Act of 1996 (HIPAA) in the US, and as such are only accessible under specific authorization of access following HIPAA guidelines. Requests to access the datasets should be directed to leanne.kaye@resmed.com.

## Ethics Statement

The studies involving human participants were reviewed and approved by Copernicus Institutional Review Board (PRH1-18-132). Written informed consent for participation was not required for this study in accordance with the national legislation and the institutional requirements.

## Author Contributions

Analyses were performed by RG. All authors contributed to the conceptualization and design of the manuscript, data interpretation, and manuscript preparation and revision.

## Conflict of Interest

LK, RG, and MB are employees of ResMed. DS and MW are employees of Propeller Health, an affiliate of ResMed. All authors receive compensation and stock as part of their employment. The authors declare that this study received funding from Propeller Health, a subsidiary of ResMed. The funder had the following involvement in the study: study design, collection, analysis, interpretation of data, the writing of this article and the decision to submit it for publication.
