# Supplementary material for: Concurrent Improvement Observed in Patient-Reported Burden and Sensor-Collected Medication Use Among Patients Enrolled in a COPD Digital Health Program
Source: Front Digit Health. 2021 Apr 9;3:624261. doi: 10.3389/fdgth.2021.624261 (PMC8521990; doi:10.3389/fdgth.2021.624261)
Supplement: Supplementary file 1 [file Table_1.DOCX]

Supplementary Material

**Supplementary Table 1. Sensitivity analysis adjusting for CAT, SABA use and adherence.**

|  | **Mean (SD) at baseline** | **Mean (SD) at 6 months** | **Estimates** | **Lower 95% CI** | **Upper 95% CI** | **P value** |
| --- | --- | --- | --- | --- | --- | --- |
| **Overall, n = 611** |  |  |  |  |  |  |
| **CAT** | 22.6 (7.8) | 21.7 (8.1) | -0.8 | -1.4 | -0.1 | 0.02 |
| **SABA use, puffs/day** | 2.3 (3.1) | 1.6 (2.5) | -0.6 | -0.8 | -0.3 | < 0.001 |
| **Adherence, %** | 81.1 (27.1) | 76.8 (29.8) | -3.5 | -6.5 | -0.6 | 0.02 |
| **High burden, n = 378** |  |  |  |  |  |  |
| **CAT** | 27.4 (4.7) | 25.4 (6.6) | -2.1 | -2.9 | -1.3 | < 0.001 |
| **SABA use, puffs/day** | 2.7 (3.4) | 1.8 (2.5) | -0.6 | -1.0 | -0.2 | < 0.01 |
| **Adherence, %** | 79.6 (27.0) | 74.5 (30.3) | -4.6 | -8.5 | -0.7 | 0.02 |
| **Low burden, n = 233** |  |  |  |  |  |  |
| **CAT** | 14.7 (4.8) | 15.6 (6.5) | 1.1 | 0.1 | 2.1 | 0.036 |
| **SABA use, puffs/day** | 1.7 (2.4) | 1.3 (2.3) | -0.5 | -0.9 | -0.2 | < 0.01 |
| **Adherence, %** | 82.8 (27.2) | 80.0 (28.8) | -3.0 | -7.8 | 1.9 | 0.23 |

*All analyses adjusted for calendar month and age.

**For analyses of CAT, sensitivity analyses also adjusted for SABA use and adherence.

For analyses of SABA use, sensitivity analyses also adjusted for CAT and adherence.

For analyses of adherence, sensitivity analyses also adjusted for CAT and SABA use.
